# Experience as a doctor in the developing world: *does it benefit the clinical and organisational performance in general practice?*

**DOI:** 10.1186/1471-2296-10-80

**Published:** 2009-12-15

**Authors:** Pieter van den Hombergh, Niek J de Wit, Frank AM van Balen

**Affiliations:** 1 University Medical Centre Utrecht, Julius Centre for Health Sciences and Primary Care, Utrecht; PO Box 85060; 3508 AB Utrecht; The Netherlands; 2 IQhealthcare, Center for Quality of Care Research, University Medical Center Nijmegen (UMCN), PO Box 9101, 6500 HB Nijmegen, The Netherlands; 3 Dutch Society for Tropical Medicine, Working Group of General Practice Care and International Health, Postbus 5032, 1200 MA, Hilversum, The Netherlands

## Abstract

**Background:**

Many physicians have medical experience in developing countries early in their career, but its association with their medical performance later is not known. To explore possible associations we compared primary care physicians (GPs) with and without professional experience in a developing country in performance both clinical and organisational.

**Methods:**

A retrospective survey using two databases to analyse clinical and organisational performance respectively. Analysis was done at the GP level and practice level.

517 GPs received a questionnaire regarding relevant working experience in a developing country. Indicators for clinical performance were: prescription, referral, external diagnostic procedures and minor procedures. We used the district health insurance data base covering 570.000 patients. Explorative secondary analysis of practice visits of 1004 GPs in 566 practices in the Netherlands from 1999 till 2001. We used a validated practice visit method (VIP; 385 indicators in 51 dimensions of practice management) to compare having experience in a developing country or not.

**Results:**

Almost 8% of the GPs had experience in a developing country of at least two years.

These GPs referred 9,5% less than their colleagues and did more surgical procedures. However, in the multivariate analysis 'experience in a developing country' was not significantly associated with clinical performance or with other GP- and practice characteristics. 16% of the practices a GP or GPs with at least two years experience in a developing country. They worked more often in group and rural practices with less patients per fte GP and more often part-time. These practices are more hygienic, collaborate more with the hospital and score better on organisation of the practice. These practices score less on service and availability, spend less time on patients in the consultation and the quality of recording in the EMD is lower.

**Conclusions:**

We found interesting differences in clinical and organisational performance between GPs with and without medical experience in developing countries and between their practices. It is not possible to attribute these differences to this experience, because the choice for medical experience in a tropical country probably reflects individual differences in professional motivation and personality. Experience in a developing country may be just as valuable for later performance in general practice as experience at home.

## Background

Many doctors consider working in developing countries as an enrichment of their professional and personal development[1-4]. The limited resources and substandard conditions highly appeal upon professional skills, creativity and problem solving capacity. Former tropical doctors often claim to benefit from this experience in their later professional career[5]. In a qualitative study fellows of an International Health Fellowship Program (IHFP) felt that participation had a positive influence on their careers. While causality cannot be inferred, the fellows demonstrated a strong preference to work with underserved populations and to be engaged in community service activities. Compared with US physicians, IHFP fellows were more likely to practice primary care and obtain MPH degrees[6]. Ramsey recommends a quantitative study to confirm his results.

Many countries have a long standing tradition of supporting health care services in developing countries by stimulating newly graduated doctors to work in rural hospitals or to assist in relief programmes. These programmes are coordinated by governmental medical relief organizations and usually last for a period of several months up to a number of years. In the Netherlands doctors who apply for working as a tropical doctor for an expected period of three years or more receive additional training after graduation. They train a year or more in surgery, obstetrics and follow a 3 month course in general tropical medicine. Their experience with non endemic diseases, transcultural medicine and cost consciousness is hoped to benefit the western health care system after their return[7,8].

Not everybody is convinced of the value of this experience for the professional performance in general practice and the experience is not always accepted as relevant. A prospective study would be nonsensical because the choice for working in a developing country is likely to set you apart. A number of reports demonstrate a positive impact of electives in international health on the clinical skills, medical practice and personal development on medical students[9-11]. but quantitative data on the associations between postgraduate medical work in developing countries and professional performance in primary care are lacking.

In a previous study in Holland GPs with experience in a developing country turned out to prescribe fewer drugs and to refer fewer patients than colleagues without this experience. The survey was small scale, and did not control for confounding factors[12].

In this study we analyse the relation between 'medical experience in developing countries' and the clinical and organisational performance of general practitioners in the Netherlands. For our exploration we tried to cover as many aspects of clinical and practice management in primary care as were available by combining the results of two studies in two large groups of practices and GPs. For convenience we often use 'tropical GP' to name a GP with professional experience in the developing world.

## Methods

In our retrospective survey we used two distinct data sets (study I: Clinical performance and Study II: practice management).

For the analysis of *clinical performance *we used the regional database from AGIS, a major Health Insurance Company including 570.000 patients and 517 GPs.

For the analysis of *organisational performance *we used the national Practice Visit database with the results of the quality assessment method VIP for GPs and their practices developed by the Centre for Quality of Care research (IQhealthcare)[13].

Data were analysed at the GP and practice level. A group practice with at least one tropical GP in the team was labeled as 'having experience in the developing world'.

The ethical committee Arnhem-Nijmegen stated that ethical approval was not required for this project.

### Clinical performance (study I)

#### Domain

We sent 517 GPs a survey asking to report on all relevant medical experience both in developed and underdeveloped countries.

#### Definition

Relevant experience in developing countries was defined as at least two years clinical work in a local hospital, with preceding internships in surgery and obstetrics.

#### Data collection

The AGIS Health database contains reimbursement data of all medical interventions on the 570.000 patients in the region. Data of group practices were not available on individual level, so we used averages on practice level. From all 519 GPs in the region we collected the 1999 data on three aspects of clinical performance: number of prescriptions, of referrals and of diagnostic tests per practice. In addition we registered relevant sociodemographic details, either practice related (urban versus rural, group practice versus single handed, list size) or GP related (age).

#### Parameters

We chose referrals to those specialties for which we expected that experience in a developing country would make a difference: internal medicine, surgery, paediatrics and physiotherapy. As for prescription we compared generally accepted indicators (antibiotics, antihypertensives and tranquillizers) as well as two drug classes that indicate the tendency to use "new" drugs (Osteoporosis and prostate volume reducing medication). Finally we calculated the number of X rays and the number of (non desk top) laboratory tests per practice.

#### Analysis

All indicators were calculated per 1000 patients for each practice. Data of the participating practices were standardized for age and gender, using the composition of the total study population as a reference. Prescription data were analysed as daily defined dosages (DDD). We compared the mean (SD) and analysed differences using Student T test (two-sided, p level, 0.05, 95% CI). We applied a multivariate analysis to assess the overall impact of tropical experience on clinical performance, controlling for sociodemographic differences. To avoid confounding by either potential correlation between the three parameter indicators, or by unbalanced distribution or chance capitalization we used linear regression analyses (General Linear Model.)[14]

### Organisational performance (study II)

#### Domain

1004 GPs in 566 practices in the Netherlands were assessed with the practice visit method VIP between 1999 and 2002[15,16]. (Appendix)

#### Definition

Tropical experience was defined as having worked for more than one year in a hospital/health centre in the developing world.

#### Data collection

The practice visit method (VIP) measures 385 indicators making up in 51 dimensions of practice management. Each dimension or scale consists of a number of items and has been confirmed in factor analysis. Cronbach's alpha was calculated to establish its internal consistency. The scores were converted to percentages; item score per total number of items in the scale (Table 1).

**Table 1:** Prescription rates (in DDD), referral rates, (numbers) and use of diagnostic tests # (GPs)

GPs without/with tropical experience		Without n = 435	With n = 37	
**Number of Prescriptions**		**mean**	**mean**	**Difference (95% CI)**
All prescriptions		515.859	477.061	38.798 (- 97.5 to 175.4)
Sub-group	Antibiotics	3.100	2.802	29 (- 252 to 849)
	Hypertensive	749	648	101 (- 190 to 393)
	Tranquillizers	12.244	12.486	242 (- 2.6 to 2.1)
	Osteoporosis	1.823	1.653	170 (- 427 to 767)
	Prostate	1.066	923	143 (- 219 to 506)
				
**Number of Referrals**				
All disciplines		588	532	**56 (9 to 102)**
Subgroup	General medicine	65	60	5 (- 3 to 14)
	Pediatrics	21	22	1 (- 4 to 3)
	Surgery	87	85	2 (- 8 to 12)
	Physiotherapy*	3.330	3.054	276 (- 81 to 633)
				
**Number of diagnostic tests**				
Total		1.464	1.290	174 (- 3 to 351)
Subgroup	Lab	789	656	133 (- 2 to 269)
	radiology	676	617	59 (- 1 to 118)

*# Absolute numbers per 1000 patients standardised for age and gender*.

* number of sessions

Instruments were questionnaires for patients, GP, practice assistant and an observer. Data collection with the VIP was part of a voluntary Quality Improvement program aimed at improving the practice management. Benchmarks are the mean score of all practices and of best practices by providing Gauss curves of each of the 51 dimensions[17].

#### Parameters

We selected 26 out of 51 aspects in the field of general practice management expected to be influenced by experience in a developing country: *Infrastructural aspect*: (emergency)facilities, hygiene, medical equipment and its use, diagnostics, patient service and organisation, *Team aspects*: delegation in disease management/prevention and collaboration with colleagues and hospital, *Aspects of Communication*: use and quality of EMD and patient information, A *spects of Quality Assurance & Safety *and - at the GP-level - *Workload and Job stress *(Table 2).

**Table 2:** Difference in score on 26 dimensions of practice management# between practices and GPs*

Dimensions of practice management; 566 practices	Number of indicators/dimension	Cron-bach's Alpha	Trop. exp 91 practices	Without trop. exp 475 practices	P-value
***I Infrastructure of the practice***					
Hygiene and facilities in treatment room	8	.37	**63,4%**	**58,4%**	**.001**
Emergency facilities	10	.60	61,8%	65%	
Advanced medical equipment in the practice	7	.49	49,4%	49,8%	
Number of ophthalmological diagnostics	7	.55	43,4%	43,9%	
Laboratory test facilities in the practice	8	.68	65,7%	62,5%	
Patient score on organization of surgeries/availability *	6	.72	**141,1**	**159,4**	**.02**
Organization of the practice	11	.56	**56,2%**	**51,1%**	**.001**
Patient wants more consultation time	1	-	**21,5%**	**19%**	**.001**
Supply of preventive care	9	.54	59,3%	61%	
					
***II Team (Delegation and collaboration)***					
No of delegated medical administrative tasks	12	.77	60,5%	61,8%	
Monitoring patients with chronic disease & prevention	11	.75	39,3%	44%	
Collaboration in GP group	11	.68	70,1%	69,3%	
Protocols on collaboration with hospital & specialists	7	.59	**61,4%**	**53,8%**	**.01**
					
***III Communication and patient records***					
Use of EMD by the GP	9	.61	**62,1%**	**65,1%**	**.04**
Quality of the EMD	4	.64	66,2%	63,9%	
Patient finds GP information in consultation adequate	3	.55	54,3%	56,2%	
					
***IV Quality improvement***					
Audit, assessment and other QI in the GP-group	8	.66	55,7%	50,8%	
Quality assurance in the practice	10	.58	38,4%	34,4%	
No. of hours/year of accredited post graduate training	1	hours	55,2%	53,5%	
					
***Jobstress (GP level)***			**111 GPs**	**893 GPs**	
Job satisfaction: pleasure, interest, commitment	4	.72	8,1	7,9	
Inappropriate demands by patients	4	.67	11,7	11,7	
Experienced workload (feeling the end of the day)	16	.93	66,0	67,7	
					
***Workload (calculated for full timers ≥ 90% fte)***			**49 GPs**	**492 GPs**	
Time spent on direct contact with patients (hrs/wk)		hours	32,6	34,4	
Total of hours per week of practice activities		hours	52,5	53,8	
Total workload in hrs/wk minus wanted workload		hours	10,1	11,3	
Time spent on QI (inc. Reading, CME etc.)		hours	3,4	3,7	

# Logistic regression correcting for single handed and rural practice

* Practices with (N = 91) or without (N = 475) and GPs with (N = 111) or without tropical experience (N = 893)

#### Analysis

Both study groups were compared to the Dutch National study for socio-demographic variables, GP and practice characteristics (Table 3). We compared workload using only results of full time working GPs; we compared job stress using results of all GPs. Experience in a developing country was entered in a regression model with the selected 26 aspects as dependent variables. In a General Linear Model we corrected for rurality and type of practice.

**Table 3:** Sociodemographic details of 371 practices and 472 GPs with and without tropical experience

	Study I Clinical data			Study II practice management		
Tropical experience	With	Without	Chi square significance	With	Without	Chi square significance
Practice level 371 practices	N = 30	N = 371		N = 91	N = 475	
- Urbanisation grade			p = 0.43			p = 0.007
0-30.000	50,0%	38,2%		63%	49%	
30.000-100.000	23,3%	31,5%		16%	32%	
> 100.000	26,7%	30,3%		21%	19%	
- Practice organisation			p = 0.04			p = 0.005
Single	30,0%	44,2%		33%	50%	
Two partner	63,3%	40,2%		26%	27%	
Group + health centre	6,7%	15,6%		41%	23%	
- List size (Fte GP/1000 pats)	0.54	0.59	p = 0.65	0.43e	0.41	p = 0.003
						
**GP level 472 GPs**	**N = 37**	**N = 435**		**N = 111**	**N = 893**	**T-test**
40%	40%	42.2%	p = 0.5	40%	42%	p = 0.7
Sexe (female)	NA	NA		18%	24%	p = 0.2
Full time	NA	NA		44%	55%	p = 0.03
Years experience as a GP	NA	NA		12,6	15,7	p = 0.048

## Results

### Clinical performance (study I)

From the 517 GPs, data of 45 GPs had to be excluded (24 GPs moved to another region, of 14 GPs the data were incomplete and one group practice of 7 GPs was excluded because only one GP had experience in a developing country). The data of the remaining 472 (91%) GPs) working in 401 practices) could be included in the analysis. In total 68 GPs (14.4%) had e professional experience in developing countries. Of these 31 GPs (6.6%) worked for a short period, either during internships, medical relief program or in a non clinical field, and 37 GPs (7.8%) had at least two years of professional postgraduate experience in a developing country. These GPs differed from their colleagues predominantly in practice set up (Appendix): only 30% (versus 44.2%) worked in a single handed practice (p = 0.043).

The prescription volume of these GPs did not differ from that of their colleagues (table 1): the absolute number of prescriptions was 7% lower but this was not significant (95% CI of the difference in prescription number; 97-751 vs 175-347 per 1000 patients). Differences for the five individual drug classes were not significant.

GPs with experience in a developing country referred less patients during 1999 as compared to their colleagues (n = 56 per 1000 patients, 95% CI: 9-102; table 1). The differences in number of referrals to the separate specialties were not significant

The total number of diagnostic tests applied during 1999 was 12% less, but neither the difference in total number of tests, nor in separate numbers for X-rays or laboratory tests were significant (table 1).

In the multivariate analysis we could not detect an overall effect of experience in a developing country on each of the three indicators of clinical performance (p = 0.202 in the linear regression model. Though also sociodemographic characteristics did not have an overall effect, effect modification occurred: younger GPs with a greater number of patients (p = 0.010) in urban areas (p = 0.031) or group practices (p = 0.043) tend to refer more and use more diagnostic tests. The percentage explained variance in the model was 23% (referral) 14% (prescription) and 28% (diagnostic test).

### Organisational performance (study II)

The 566 practices in this dataset were representative for the Dutch situation (NIVEL 2001, table 3); group practices were slightly overpresented and single handed practices underpresented. The GPs with experience in a developing country worked more often in group practices and more often in rural and dispensing practices, had less listed patients and had more than average practice assistance.

Of these practices 91 (16%) had one or more GPs with experience in developing countries. Table 2 shows the comparison between practices with or without a tropical GP on the 26 aspects of practice management. The 91 practices with a tropical GP did not differ significantly from other practices on all but 6 of the 26 selected dimensions.

After correction for rurality and type of practice practices with a tropical GP scored higher on 'Hygiene and facilities in treatment room', on 'Organisation of the practice' and on 'Protocols on collaboration with hospital & specialists' but lower on 'Patient wants more consultation time', on 'Patient's score on organisation of the surgery, on availability of the GP' and on 'Use of EMD by the GP' (Table 3). No difference was found in Equipment, Delegation, Organisation of Quality, Workload and Job stress.

The practice with a tropical GP has not more general or more emergency equipment, does not perform more therapeutic or diagnostic tasks and does not delegate more tasks to the practice assistant.

Full time working tropical GPs work two hours less than their colleagues, but this is not significant nor the finding that they feel a little more tired at the end of the day.

## Discussion

This is the first report with quantative data on the associations between professional experience in the developing world and the professional performance of general practioners after repatriating. We found some differences in clinical and organizational performance between GPs with and without former experience in developing countries. Most importantly, we could not confirm a lower number of prescriptions, referrals and diagnostic tests nor did practices differ in equipment and delegation of tasks to the practice assistant.

Because it is possible that the effect of having worked in developing countries diminishes over time, we examined whether age of the GP affected the outcome of clinical performance, but no effect was found.

We did find some positive associations. 'Tropical GPs' were found more often in group practices, in rural areas and had less patients/fte GP. This does not surprise since these doctors used to work in rural hospitals in developing countries.

Practices with tropical GPs perform better on 'Organisation of the practice', on Hygiene and on 'Collaboration with the hospital and specialists'. 'Organisation of the practice' consists of 11 items like e.g. the practice has a practice leaflet, has an internal lab form, has practice meetings with minutes, has protocols, etc. Tropical GPs may value organization better and may be more hospital oriented because of their longer stay in hospitals during their tropical medicine training. It is remarkable that patients in practices with tropical GPs are less happy with the service and availability of the GP and with the consultation time. The quality of their medical record keeping in the EMD is also less complete. That may be due to another perception of urgency and to keeping more distance to patient, being a known attitude change of working in hospitals.

### Strengths and limitations of the study

We used data from two large and representative general practice databases collected with well-developed validated instruments. In spite of this power a limitation is that the study is retrospective and that it took a long time to gather and analyse the data. The data from the district health insurance data base consisted of only sick fund patients, but in the Dutch Health Care system this selection is not likely to have affected the results. In our analysis of prescription rates we measured DDD (Defined Daily Dosage) and did not look at the indication for the prescriptions.

Analysis at practice level in case of group practices may have diluted differences in organisational performance, meaning that the differences may be more substantial.

The large number of indicators (n = 385) regarding the practice management may have increased the chance of finding accidentally significant differences.

Our correction for rurality and type of practice in the analysis is questionable. Tropical GPs choose to work in a different setting as part of previous experience on what suits them best. If the tropical experience leads to that choice one could question if correction for this in the analysis is appropriate.

### Implications

An explanation for the relatively minor differences in clinical performance is that both tropical and regular GPs become more experienced over the years, with both career paths contributing to performance. The different choices in type of practice and in priorities in management and care can be the consequence of career preferences of the GP as well as the impact of experience in a developing country. The more 'medical/clinical' orientation of the tropical GP can be due to longer hospital training and/or to working in a tropical hospital and that orientation has yet to prove its edge.

## Conclusion

The relation between working experience in developing countries and performance in general practice has become more clear. Experience in developing countries after graduation does not result in substantial differences in general practice performance compared to primary care physicians without such career. Yet, one would like to have a more detailed understanding of the relation to know what to promote in professional training of medical professionals. Our results support the present educational policy in medical training that medical experience in developing countries is probably as valuable as experience at home.

## Competing interests

The authors declare that they have no competing interests.

## Authors' contributions

PH was responsible for the second study and integrated both studies to answer the research question. He also coordinated the analysis and the successive versions of the manuscript. NJW carried out the first study, analysed the data helped to develop the research questions and wrote the introduction and conclusions. FAMB helped in the analysis and participated in all discussions and draft of the manuscript. All authors read and approved the final manuscript.

## Appendix

### The Setting and Management of Dutch General Practice (2001)

In total 7170 GPs (= 1 GP/2274 patients; ♂ = 79%, ♀ = 21%)

Single-handed 40%; Dispensing GPs 8%, GP-trainers 14%, duo-practice 34%, group practice 25%.

The GP has a gate keeping role referring only 6% of all health problems presented to the GP.

GP (locum) groups coordinate emergency care (7 × 24 hrs), home care, cooperation and QI.

The practice assistant works partly as a receptionist and partly as a practice nurse.

## Pre-publication history

The pre-publication history for this paper can be accessed here:

http://www.biomedcentral.com/1471-2296/10/80/prepub
